# Ethnic differences in risk factors for obesity in New Zealand infants

**DOI:** 10.1136/jech-2014-204464

**Published:** 2015-03-20

**Authors:** Laura D Howe, Lis Ellison-Loschmann, Neil Pearce, Jeroen Douwes, Mona Jeffreys, Ridvan Firestone

**Affiliations:** 1 MRC Integrative Epidemiology Unit at the University of Bristol, University of Bristol, Bristol, UK; 2 School of Social and Community Medicine, University of Bristol, Bristol, UK; 3 Centre for Public Health Research, Massey University, Wellington, New Zealand; 4 London School of Hygiene and Tropical Medicine, London, UK

## Abstract

**Background:**

In New Zealand, the burden of childhood obesity is greatest in Māori and Pacific children.

**Methods:**

In 687 infants from an internet-based birth cohort in New Zealand, we investigated ethnic differences in early life risk factors for later obesity, the degree to which these were explained by sociodemographic factors, and the extent to which ethnic differences in weight at age 3 months were explained by measured risk factors.

**Results:**

The risk of having an obese mother was double in Māori and Pacific infants compared with NZ European infants (prevalence 24% and 14%, respectively; OR 2.23, 95% CI 1.23 to 4.04). Māori and Pacific infants had higher weights in the first week of life and at 3 months (mean difference 0.19 kg, 95% CI 0.01 to 0.38), and their mothers had higher scores on a ‘snacks’ dietary pattern and lower scores on ‘healthy’ and ‘sweet’ dietary patterns. These inequalities were not explained by maternal education, maternal age or area-based deprivation. No ethnic differences were observed for maternal pre-pregnancy physical activity, hypertension or diabetes in pregnancy, exclusive breastfeeding or early introduction of solid foods. Ethnic inequalities in infant weight at 3 months were not explained by sociodemographic variables, maternal pre-pregnancy body mass index or dietary pattern scores or by other measured risk factors.

**Conclusions:**

This study shows excess prevalence of early life risk factors for obesity in Māori and Pacific infants in New Zealand and suggests an urgent need for early interventions for these groups.

## Introduction

The past few decades have seen a dramatic rise in the prevalence of overweight and obesity in most high-income countries, even in very young children.^1^ In New Zealand, one in nine children (11%) aged between two and 14 years are obese, and a further one in five (22%) are overweight with the greatest burden observed in the indigenous Māori population and people from the Pacific Islands. Compared with the overall population-wide prevalence of childhood obesity of 11%, the prevalence in Māori children is 19% and in Pacific children it is 27%.^2^

Being overweight or obese as a child is associated not only with short-term and long-term health risks, particularly for cardiovascular health^3^ and type 2 diabetes,^4^ but also for mental health^5^ and social outcomes such as educational attainment.^6^ There is now strong evidence that obesity often begins early in life^7^ ^8^ and that prenatal and early-life factors,^7^ ^9^ including low socioeconomic position,^10^ ^11^ maternal obesity,^12^ ^13^ maternal diet during pregnancy,^13^ maternal hypertension or diabetes,^14^ no or short duration of breastfeeding,^15^ ^16^ early introduction of solid foods^15^ and greater weight in early infancy,^17–19^ are associated with childhood obesity. Furthermore, despite some interventions with established effectiveness,^20–22^ adult obesity has proved very difficult to treat,^21^ ^23^ and obesity established in early life tends to persist across the life course,^24^ emphasising the need for early preventive interventions. Understanding ethnic differences in early life risk factors for later obesity may thus help to inform strategies aimed at preventing ethnic inequalities in obesity later in life.

This study examined ethnic differences in established early life risk factors for obesity in a prospective New Zealand cohort study of 3-month-old infants to assess whether these ethnic differences persist after adjustment for sociodemographic variables, and to examine the extent to which ethnic differences in infant weight at age 3 months are explained by other risk factors.

## Methods

### The Early Life Factors Study

The Early Life Factors (ELF) study is an internet-based birth cohort study investigating the associations between ELF including environmental exposures and a wide range of health outcomes including obesity. The primary location for the ELF cohort is Wellington, New Zealand, while additional study sites are located in other main cities (Christchurch and Auckland). Pregnant women aged 16 and above were eligible to participate. The main source of recruitment of pregnant women was at ‘parent and child shows’ marketed at expecting and experienced parents. The shows occur annually in the main urban centres (Auckland, Wellington and Christchurch). Additional recruitment methods included information in: prenatal booklets, antenatal clinics within hospitals and sonography practices, and participants who enrolled through an internet engine search. A total of 2197 pregnant women indicated their willingness to participate from September 2008 to September 2011. Almost half were from Wellington (45%), with the others mostly from Auckland (33%) and Canterbury (12%). Of those, 1042 (44%) have actively contributed data, with reasons for non-participation including loss to follow-up (81%), later refusal to participate (12%), miscarriage, non-viable pregnancy or death of the baby, and moving to another country. Participants were asked to complete one questionnaire in late pregnancy (median 30.5 weeks gestation, IQR 23–36 weeks), and a second questionnaire when their child was approximately 3 months old. Participants could choose to complete the questionnaire over the internet or use a paper-based postal copy.

### Assessment of child's ethnicity

Mothers reported the ethnicity of their child in the 3 month questionnaire. Prioritised self-identified ethnicity is standard health research practice in New Zealand.^25^ If Māori was selected as one of the multiple potential responses, the child was classified as Māori. Respondents selecting one or more of several Pacific Island ethnicities (but not selecting Māori) were prioritised as Pacific. Participants who selected New Zealand European as their ethnicity were classified as NZ European (hereafter referred to as ‘European’). Owing to the small number of Pacific Island infants, we grouped these with Māori infants, since Māori and Pacific infants are more similar than Pacific and European infants in terms of shared risk factors, and both ethnicities have a higher prevalence of maternal and childhood obesity.^26^ ^27^ Also, owing to the small numbers within each group, all other ethnicities (predominantly Chinese and Indian) were classified as ‘other’.

### Assessment of early life risk factors for obesity

In the pregnancy questionnaire, women reported their age, educational level, height and pre-pregnancy weight and usual dietary intake via a number of questions about the frequency of consumption of certain foods (full questionnaire reproduced in online only supplementary material), and whether before their pregnancy they regularly undertook at least 1 h per week of physical activity sufficiently vigorous to make them sweat. We dichotomised maternal education as a university degree or higher versus less than a degree. Body mass index (BMI) was calculated as weight in kilograms divided by height in metres squared, from which we defined overweight as BMI≥25 kg/m^2^ and <30 kg/m^2^ and obesity as BMI≥30 kg/m^2^ since these categories are used in clinical practice; the same definitions of overweight and obesity were used in all ethnic groups. Maternal age was categorised as <25 years, 25 to <25 years and ≥35 years.

In the 3-month questionnaire, mothers reported whether they experienced hypertension or diabetes during pregnancy, the child's weight within the first week of life and at age 3 months (mothers were asked to copy these from the child health record, which is given to parents of all newborns in New Zealand), whether they exclusively breastfed the infant in the first 3 days of life and still at age 3 months (defined as breast milk being the only food or drink given to the infant) and whether they had introduced the infant to solid food before 3 months. We grouped pre-existing and pregnancy-onset hypertension and diabetes due to the small numbers.

The New Zealand Deprivation Index 2006^28^ was used to calculate an area-based measure of deprivation based on the mother's address during pregnancy.

### Statistical analysis

The analysis of dietary patterns was carried out using principal component analysis (PCA),^29–31^ an approach which seeks to create meaningful summary ‘patterns’ that describe types of diet. Each dietary pattern has ‘weights’ for each food type, and these weights were used to calculate a score for each dietary pattern for each woman; higher scores for a given dietary pattern indicate that the woman's diet was more closely matched to that dietary pattern. Note that each woman is assigned a score for each dietary pattern, since it is possible (and likely) that a given woman's diet will have characteristics of more than one pattern; thus, the dietary pattern scores are a continuous measure of how closely a woman's diet matches each ‘type’ of diet. PCA analysis was carried out in Stata using the pca command, with orthogonal rotation to improve interpretability of the components. The dietary pattern scores were standardised to have a mean of zero and a variance of one. We included in the PCA data on frequency of intake of multiple food types (boiled vegetables, stir fry vegetables, frozen vegetables, fresh fruits, oats, pulses, cheese, pies, sausages, burgers, pizza, chips, BBQ foods, fried foods, rice, pasta, pudding, cake, chocolate bars, chocolate, sweets, potato crisps and fizzy drinks) as well as binary variables indicating whether white bread and brown bread were eaten on most days. Consumption of other food types was also reported (full details in the online only supplementary material), but the addition of further food types to our analysis reduced the proportion of variance explained by the first few principal components (patterns) and resulted in less interpretable dietary patterns. The number of principal components/dietary patterns selected for further analysis was selected on the basis of a scree plot, which describes the proportion of variance in the indicator variables explained by each principal component.

The association between child's ethnicity and sociodemographic variables (maternal education and age, and New Zealand Deprivation Index) was assessed using logistic or multinomial logistic regression as appropriate. Associations between ethnicity and other early life risk factors for later obesity were evaluated using linear, logistic or multinomial logistic regression as appropriate, with and without adjustment for sociodemographic variables.

The degree to which ethnic differences in infant weight were explained by other factors was examined using linear regression, with adjustment for potential mediators. For all regression models, residuals were approximately normally distributed.

To minimise selection bias^32^ and increase efficiency, multivariate multiple imputation^33^ was carried out to impute missing data on early life risk factors. Multivariate multiple imputation creates a specified number of copies of the data (in our case, 20 copies) in which missing values are imputed by chained equations, with an appropriate level of randomness. Results are obtained by averaging the results from analysis of each of these 20 data sets using Rubin's rules.^34^ In this procedure, the SEs for any regression coefficients (used to calculate p values and 95% CIs) take account of the uncertainty in the imputations as well as uncertainty in the estimate. We included in our multivariate multiple imputation analysis all participants for whom we had data on the child's ethnicity from the 3-month questionnaire, and used all variables included in our analyses to impute all others. We present the results from the multivariate multiple imputation analysis as our main results, and results from the analysis of participants with complete observed data on all variables of interest as online only supplementary material.

## Results

Data on the infant's ethnicity were available for 687 participants (67% of those who have contributed any data), who form the eligible sample for our multivariate multiple imputation analysis. Of these 687 infants, 493 (71.8%) were of European ethnicity, 21 (3.1%) were Pacific, 94 (13.7%) were Māori and 79 (11.5%) were of other ethnicity. Thus, 16.7% of our participants were of Māori or Pacific ethnicity (table 1). Most of the mothers were aged 25 to <35 years, and most were educated to university degree level (table 1).

**Table 1:** Participant characteristics

	Prevalence or mean (SD) by ethnic group		
	European N=493	Māori or Pacific N=115	Other N=79
Sociodemographic variables			
Mother educated to less than University degree level	15.2%	27.4%	3.3%
Mother's age, years			
<25	6.4%	25.3%	2.5%
25 to <35	63.7%	60.4%	64.8%
≥35	30.0%	14.4%	32.7%
NZ deprivation index 2006			
1 (lowest deprivation)	28.8%	16.4%	21.5%
2	29.2%	20.4%	32.9%
3	20.1%	18.4%	17.7%
4	12.9%	30.4%	20.5%
5 (highest deprivation)	9.0%	14.4%	7.5%
Maternal pre-pregnancy BMI, kg/m^2^			
<25	59.6%	45.6%	64.1%
25 to <30	26.3%	30.3%	29.3%
≥30	14.1%	24.2%	6.7%
Mother did physical activity pre-pregnancy	82.2%	83.9%	86.2%
Mother's diet in pregnancy*			
Processed foods (SD)	−0.02 (1.01)	0.16 (0.97)	−0.13 (0.96)
Snacks (SD)	−0.03 (0.98)	0.33 (1.03)	−0.28 (0.98)
Healthy foods (SD)	0.03 (0.99)	−0.20 (1.06)	0.10 (0.94)
Sweet foods (SD)	0.05 (0.99)	−0.20 (1.05)	−0.01 (0.93)
Starchy foods (SD)	−0.07 (1.01)	0.09 (0.98)	0.27 (0.92)
Hypertension in pregnancy	12.2%	16.5%	11.4%
Diabetes in pregnancy	3.7%	2.6%	6.3%
Infancy characteristics			
Weight in first week (kg)	3.51 (0.56)	3.62 (0.68)	3.45 (0.51)
Weight at 3 months (kg)	6.02 (0.85)	6.21 (1.01)	6.07 (0.89)
Exclusive breastfeeding in first 3 days	80.4%	77.4%	79.1%
Exclusive breastfeeding at 3 months	52.6%	51.3%	59.4%
Solid foods introduced by 3 months	29.6%	35.7%	24.2%

Values are percentages or mean (SD) for 687 participants using imputed data.

*Dietary pattern scores are standardised to have a mean of zero and a variance of one; values in this table can therefore be interpreted as SEs from the overall population mean.

BMI, body mass index.

Distributions of all variables were similar in the observed data and in the 20 data sets created by multivariate multiple imputation (see online supplementary table S1). Complete data on all variables of interest were available for 422 participants; no strong ethnic differences were observed between participants with fully observed data and those with some missing data, but mothers who did not provide complete data were more likely to be educated to less than degree level and to live in an area of higher deprivation (see online supplementary table S2).

Principal components analysis of the mother's dietary intake data indicated that five principal components provided a reasonable summary of dietary patterns, with the elbow of the Screeplot being approximately at 5 (see online supplementary figure S1) and the first five components cumulatively explaining approximately 40% of the variance in the dietary intake data and resulting in interpretable dietary patterns (interpretation of higher order components was less clear; see online supplementary table S3). The weightings assigned to each of these five dietary patterns indicate that the following are suitable descriptions of the five dietary patterns: (1) processed foods (characterised by high intake of burgers, pizza, pies, chips and fried foods), (2) snacks (characterised by high intake of chocolate, sweets, fizzy drinks and crisps), (3) healthy foods (characterised by high intake of stir fried vegetables, fresh fruit, oats and pulses), (4) sweet foods (characterised by high intake of biscuits and cake), (5) starchy foods (characterised by high intake of rice and pasta; see online supplementary table S3).

Māori and Pacific infants were more likely to have a mother who was educated to less than university degree level, who was younger than 25 years old, or to live in an area of high deprivation (table 2). Infants of ‘other’ ethnicity were less likely to have a mother who was educated to less than degree level, but were slightly more likely to live in an area of high deprivation (table 2).

**Table 2:** Association between infant's ethnicity and sociodemographic variables

	European N=493	Māori or Pacific N=115	Other N=79
Mother educated to less than University degree level	1 (ref)	2.10 (1.16 to 3.80) p=0.01	0.19 (0.05 to 0.73) p=0.02
Mother's age, years			
<25	1 (ref)	4.20 (2.13 to 8.29) p<0.001	0.33 (0.05 to 2.42) p=0.28
25 to <35	1 (ref)	1 (ref)	1 (ref)
≥35	1 (ref)	0.50 (0.26 to 0.98) p=0.05	1.07 (0.62 to 1.85) p=0.81
NZ deprivation index 2006			
1 (lowest deprivation)	1 (ref)	1 (ref)	1 (ref)
2	1 (ref)	1.23 (0.56 to 2.68) p=0.61	1.51 (0.74 to 3.11) p=0.61
3	1 (ref)	1.61 (0.76 to 3.40) p=0.21	1.18 (0.51 to 2.75) p=0.70
4	1 (ref)	4.16 (2.05 to 8.43) p<0.001	2.14 (0.94 to 4.84) p=0.07
5 (highest deprivation)	1 (ref)	2.81 (1.19 to 6.63) p=0.02	1.11 (1.19 to 6.33) p=0.86

**N**=687 using multiply imputed data.

Values are ORs (95% CIs) comparing Māori and Pacific children and other ethnicity children to European children. Logistic regression was used for maternal educational level, with ‘more than University degree’ as the reference category. Multinomial logistic regression was used for maternal age and NZ deprivation index, with 25 to <35 years and lowest deprivation quintile used as the reference categories.

Māori and Pacific infants were twice as likely as European infants to have a mother who was obese (OR 2.23, 95% CI 1.23 to 4.04; table 3). The prevalence of maternal obesity was 23.8% in Māori and Pacific women, 14.1% in European women and 6.2% in women of other ethnicities. Ethnic differences in overweight were less pronounced, with the prevalence being 30% in Māori and Pacific women, 26.3% in European women and 29.2% in women of other ethnicity (table 1).

**Table 3:** Association between infant's ethnicity and known risk factors for obesity (unadjusted)

	European N=493	Māori or Pacific N=115	Other N=79
Categorical risk factors; ORs and 95% CIs			
Maternal pre-pregnancy BMI, kg/m^2^			
<25	1 (ref)	1 (ref)	1 (ref)
25 to <30	1 (ref)	1.51 (0.87 to 2.62) p=0.15	1.04 (0.59 to 1.82) p=0.90
≥30	1 (ref)	2.23 (1.23 to 4.04) p=0.01	0.43 (0.15 to 1.24) p=0.12
Mother did physical activity pre-pregnancy	1 (ref)	1.14 (0.61 to 2.12) p=0.68	1.36 (0.64 to 2.90) p=0.42
Hypertension in pregnancy	1 (ref)	1.43 (0.81 to 2.50) p=0.21	0.93 (0.44 to 1.95) p=0.84
Diabetes in pregnancy	1 (ref)	0.70 (0.20 to 2.43) p=0.58	1.78 (0.64 to 4.93) p=0.27
Exclusive breastfeeding in first 3 days	1 (ref)	0.84 (0.51 to 1.37) p=0.47	1.20 (0.70 to 2.06) p=0.41
Exclusive breastfeeding at 3 months	1 (ref)	0.95 (0.63 to 1.42) p=0.80	1.32 (0.81 to 2.13) p=0.27
Solid foods introduced by 3 months	1 (ref)	1.32 (0.86 to 2.03) p=0.20	0.76 (0.44 to 1.32) p=0.34
Continuous risk factors; mean differences and 95% CIs			
Mother's diet in pregnancy*			
Processed foods (SD)	0 (ref)	0.17 (−0.08 to 0.43) p=0.19	−0.11 (−0.38 to 0.15) p=0.39
Snacks (SD)	0 (ref)	0.36 (0.13 to 0.59) p=0.002	−0.25 (−0.52 to 0.01) p=0.06
Healthy foods (SD)	0 (ref)	−0.23 (−0.49 to 0.03) p=0.09	0.07 (−0.22 to 0.36) p=0.63
Sweet foods (SD)	0 (ref)	−0.25 (−0.51 to 0.01) p=0.06	−0.07 (−0.33 to 0.20) p=0.63
Starchy foods (SD)	0 (ref)	0.16 (−0.06 to 0.38) p=0.16	0.33 (0.05 to 0.62) p=0.02
Child's weight in first week (kg)	0 (ref)	0.11 (−0.01 to 0.23) p=0.07	−0.06 (−0.20 to 0.08) p=0.42
Child's weight at 3 months (kg)	0 (ref)	0.19 (0.01 to 0.38) p=0.04	0.05 (−0.16 to 0.27) p=0.62

N=687 using multiply imputed data.

Values are ORs or mean differences (95% CIs) comparing Māori or Pacific children and other ethnicity children to European children. Multinomial logistic regression was used for maternal pre-pregnancy BMI, with <25 kg/m^2^ as the reference category. Logistic regression was used for binary risk factors, and linear regression for continuous risk factors. Sociodemographic variables included in the analyses were maternal age, maternal education and NZ deprivation index.

*Dietary pattern scores are standardised to have a mean of zero and a variance of one; values in this table can therefore be interpreted as SDs from the overall population mean.

BMI, body mass index.

There was no evidence of ethnic differences in maternal pre-pregnancy physical activity, hypertension or diabetes in pregnancy, exclusive breastfeeding or early introduction of solid foods (table 3). Mothers of Māori and Pacific children tended to have a higher score on the snacks dietary pattern as compared with mothers of European children (mean difference 0.36 SD, 95% CI 0.13 to 0.59) and a lower score on the healthy foods dietary pattern (mean difference −0.23, 95% CI −0.49 to 0.03) but lower scores on the sweet foods dietary pattern (mean difference −0.25 SD, 95% CI −0.51 to 0.01; table 3). Māori and Pacific children had on average a higher weight both in the first week of life (mean difference compared with European children 0.11 kg, 95% CI −0.01 to 0.23) and at age 3 months (mean difference compared with European children 0.19 kg, 95% CI 0.01 to 0.38; table 3). There was no evidence of differences between the European and ‘other’ ethnic groups in any early life risk factor (table 3).

Inequalities between Māori and Pacific infants and European infants in terms of maternal obesity, maternal diet in pregnancy and weight in early life did not attenuate after adjustment for maternal age, education level and area-level deprivation (table 4). The magnitude of ethnic differences in infant weight at age 3 months remained similar after adjustment for all measured potential mediators (sociodemographic variables, maternal pre-pregnancy BMI, maternal dietary pattern scores, hypertension during pregnancy, diabetes during pregnancy, physical activity during pregnancy, exclusive breastfeeding at 3 days, exclusive breastfeeding at 3 months and early introduction of solid foods), although the CI was wider and spanned the null value after adjustment for potential mediators (table 5).

**Table 4:** Association between child's ethnicity and known risk factors for later obesity (with adjustment for sociodemographic characteristics)

	European N=493	Māori or Pacific N=115	Other N=79
Categorical risk factors; ORs and 95% CIs			
Maternal pre-pregnancy BMI, kg/m^2^			
<25	1 (ref)	1 (ref)	1 (ref)
25 to <30	1 (ref)	1.47 (0.80 to 2.68) p=0.21	1.03 (0.58 to 1.82) p=0.92
≥30	1 (ref)	2.27 (1.21 to 4.26) p=0.01	0.44 (0.15 to 1.29) p=0.14
Mother did physical activity pre-pregnancy	1 (ref)	1.79 (0.92 to 3.48) p=0.09	1.30 (0.60 to 2.81) p=0.50
Hypertension in pregnancy	1 (ref)	1.50 (0.82 to 2.75) p=0.19	0.97 (0.45 to 2.07) p=0.93
Diabetes in pregnancy	1 (ref)	0.74 (0.20 to 2.73) p=0.65	1.70 (0.59 to 4.91) p=0.32
Exclusive breastfeeding in first 3 days	1 (ref)	0.72 (0.43 to 1.23) p=0.23	0.95 (0.52 to 1.74) p=0.87
Exclusive breastfeeding at 3 months	1 (ref)	0.98 (0.63 to 1.52) p=0.92	1.24 (0.75 to 2.03) p=0.40
Solid foods introduced by 3 months	1 (ref)	1.24 (0.77 to 1.98) p=0.38	0.80 (0.45 to 1.40) p=0.43
Continuous risk factors; mean differences and 95% CIs			
Mother's diet in pregnancy*			
Processed foods (SD)	0 (ref)	0.07 (−0.18 to 0.33) p=0.57	−0.11 (−0.37 to 0.15) p=0.41
Snacks (SD)	0 (ref)	0.37 (0.12 to 0.62) p=0.004	−0.27 (−0.53 to −0.01) p=0.05
Healthy foods (SD)	0 (ref)	−0.11 (−0.37 to 0.15) p=0.40	−0.02 (−0.30 to 0.26) p=0.90
Sweet foods (SD)	0 (ref)	−0.20 (−0.46 to 0.06) p=0.12	−0.09 (−0.36 to 0.17) p=0.50
Starchy foods (SD)	0 (ref)	0.23 (−0.00 to 0.47) p=0.05	0.30 (0.02 to 0.58) p=0.03
Child's weight in first week (kg)	0 (ref)	0.08 (−0.04 to 0.21) p=0.20	−0.05 (−0.19 to 0.10) p=0.53
Child's weight at 3 months (kg)	0 (ref)	0.16 (−0.04 to 0.36) p=0.11	0.07 (−0.15 to 0.29) p=0.65

**N**=687 using multiply imputed data.

Values are ORs or mean differences (95% CIs) comparing Māori or Pacific children and other ethnicity children to European children. Multinomial logistic regression was used for maternal pre-pregnancy BMI, with <25 kg/m^2^ as the reference category. Logistic regression was used for binary risk factors, and linear regression for continuous risk factors.

*Dietary pattern scores are standardised to have a mean of zero and a variance of one; values in this table can therefore be interpreted as SDs from the overall population mean.

BMI, body mass index.

**Table 5:** Association between ethnicity and infant weight at age 3 months, and the degree to which this is explained by other risk factors

	Mean difference (95% CI) in infant weight at 3 months (kg)		
	European N=493	Māori or Pacific N=115	Other N=79
Unadjusted	0 (ref)	0.19 (0.01 to 0.38) p=0.04	0.05 (−0.16 to 0.27) p=0.62
Adjusted for sociodemographic variables*	0 (ref)	0.16 (−0.04 to 0.36) p=0.11	0.07 (−0.15 to 0.29) p=0.65
Adjusted for maternal pre-pregnancy BMI	0 (ref)	0.18 (−0.01 to 0.37) p=0.06	0.06 (−0.15 to 0.28) p=0.58
Adjusted for maternal dietary pattern scores†	0 (ref)	0.22 (0.03 to 0.41) p=0.02	0.03 (−0.18 to 0.25) p=0.76
Adjusted for all measured potential mediators^‡^	0 (ref)	0.17 (−0.03 to 0.38) p=0.10	0.03 (−0.19 to 0.25) p=0.76

N=687 using multiply imputed data.

Values are mean differences (95% CIs) in infant weight at age 3 months (kg) from linear regression, comparing each ethnic group with the reference category, European ethnicity.

*Adjusted for maternal education, maternal age and NZ deprivation index.

†Adjusted for scores for all five dietary patterns.

‡Adjusted for maternal education, maternal age, NZ deprivation index, maternal pre-pregnancy BMI, scores for all five dietary patterns, hypertension during pregnancy, diabetes during pregnancy, physical activity during pregnancy, exclusive breastfeeding at 3 days, exclusive breastfeeding at 3 months, and early introduction of solid foods.

BMI, body mass index.

The results of analyses of the 422 participants with complete data on all variables of interest were broadly similar to those from our analysis using multivariate multiple imputation, although CIs tended to be wider due to lower statistical power; since some variable CIs did not include the null in analysis of the imputed data but did include the null in the complete case analysis (see online supplementary tables S4–S7). Analysis of the potential role of mediators in the association between ethnicity and infant weight at 3 months is difficult in the complete case analysis, since the CI in unadjusted analysis spans the null value (see online supplementary table S7).

## Discussion

In this study, infants of Māori and Pacific ethnicity were more likely than European infants to have obese mothers and mothers whose diet was characterised by higher scores on a ‘snacks’ dietary pattern (characterised by the high intake of chocolate, sweets, fizzy drinks and crisps) and lower scores on a ‘healthy’ dietary pattern (characterised by the high intake of stir fried vegetables, fresh fruit, oats and pulses). Māori and Pacific infants already had higher weight in the first week of life and at 3 months compared with European children. These ethnic inequalities in early life risk factors for obesity were not explained by measured sociodemographic factors.

Ethnic identity is an important axis of health inequalities in New Zealand with the health status of Māori and Pacific people being markedly poorer than that of the European population. Obesity is an area of particularly striking ethnic inequalities, with the prevalence of obesity in adult women being 80% higher in Māori and more than double in Pacific women compared with Europeans.^2^ Given the early-life origins of obesity,^35^ developing interventions to prevent obesity in childhood for these ethnic groups is a key public health strategy.

Our finding of greater weight in infancy in Māori and Pacific children is in agreement with previous research. For example, one study found that the risk of having a child with a birth weight of 4 kg or more was more than twofold higher in Pacific women compared with European women^36^ and a cohort of 722 Pacific children in New Zealand found high birth weight and rapid growth in the first years of life.^37^ Thus, the ethnic differentiation in weight trajectories in New Zealand begins in very early life.

In our study, Māori and Pacific mothers had higher scores than European women on the ‘snack’ dietary pattern, and lower scores on the ‘healthy’ and ‘sweet’ patterns, but no difference on the ‘processed’ dietary pattern. This differs somewhat from a previous study of 1714 pregnant women in which Māori and Pacific mothers were found to have higher scores on a ‘junk food’ dietary pattern,^38^ and a study of 115 pregnant women in Wellington, New Zealand, which found that Māori women consumed more sucrose than European women, and Pacific women consumed more starch.^39^ However, all three studies do show adverse dietary intakes in Māori and Pacific women. Although widely discussed as a protective factor for obesity, studying the effects of breastfeeding on child growth is fraught with methodological problems, including considerable residual confounding and reverse causality.^40^ Recently, evidence from study designs better able to address these methodological issues have questioned the causality of breastfeeding as a protective factor for later obesity;^41^ ^42^ nevertheless, breastfeeding is beneficial for other infant outcomes, including cognitive development,^42^ and it is reassuring that in this study we do not find strong ethnic differences in the prevalence of exclusive breastfeeding in the first 3 days of life and at 3 months.

The key strengths of this study include the availability of a broad range of potential early life risk factors for obesity, and the use of multivariate multiple imputation to minimise selection bias and maximise efficiency. Data on infant weight were extracted by the mothers from the child's health records; such data have been shown in other contexts to have a reasonable degree of accuracy.^43^ The self-reported nature of data on pre-pregnancy BMI is a limitation, since it is known that such data can be inaccurate, with greater degrees of inaccuracy observed in overweight people.^44^ However, evidence suggests reasonable concordance between overweight status defined on self-reported or measured BMI,^44^ including no ethnic differences in the degree of concordance in a New Zealand population,^45^ so we consider that this is unlikely to have strongly biased our results. However, the fact that ethnic differences in infant weight at age 3 months are not explained by adjustment for potential mediators may be at least partially explained by measurement error in the self-reported data. Residual confounding is also a possibility; our adjustment of ethnic differences for socioeconomic factors is unlikely to have fully accounted for the socioeconomic differences between the ethnic groups. We have not adjusted infant weight for length, and as such our estimated ethnic differences in infant weight may be confounded by length. However, the method of adjustment of infant weight for length is controversial and dependent on gestational age,^46^ and both weight-adjusted and length-adjusted weight during infancy are known to be associated with later obesity.^47^ Our earliest measure of infant weight in this sample is from 1 week, which we believe is likely to be a reasonable approximation of birth weight. Our sample size was not sufficient to assess any differences between Māori and Pacific infants. Even grouping these ethnicities, our power was limited; for example, for the difference in child's weight at 3 months between Māori and Pacific infants and European infants (0.19 kg), our sample size provided 55% power to detect this difference at the 5% significance level in the imputed data. Furthermore, we did not have sufficient numbers of infants to further explore the ‘other’ ethnic group, which had better outcomes than NZ European infants for many of the risk factors examined in our study. Small numbers of women with hypertension and diabetes in our study meant that we had limited statistical power to examine these outcomes. The women participating in this cohort were of relatively high socioeconomic position; over 25% of them were from the bottom fifth of area-level deprivation, and less than 10% were from the most deprived fifth of the population. The distribution of area-level deprivation within the participants of the cohort who are of European ethnicity broadly matches that seen in the total New Zealand population;^48^ however compared with the full New Zealand population, the cohort participants of Māori and Pacific ethnicity were relatively socioeconomically advantaged.^48^ This is likely to mean that the ethnic inequalities in risk factors observed within this study are underestimates of the true degree of inequality. Further research is necessary to understand the factors contributing to the ethnic inequalities we observe; residual confounding by socioeconomic factors, behavioural differences and genetic factors are all possibilities.

In conclusion, this study provides evidence of excess prevalence of early life risk factors for obesity, in particular maternal obesity, poor maternal diet during pregnancy and high weight in infancy in Māori and Pacific infants in New Zealand. Given the striking ethnic inequalities in obesity in childhood and adulthood in New Zealand, this suggests an urgent need for preventive interventions, including those focusing on pre-pregnancy, pregnancy and early infancy.

**What is already known on this subject?**

- In New Zealand, the burden of childhood obesity is greatest in Māori and Pacific children. Several early life (pre-pregnancy, pregnancy, perinatal and infancy) factors are known to be associated with obesity in later life. Our study examined ethnic inequalities in these early life risk factors in a cohort of 3-month-old infants in New Zealand.

**What this study adds?**

- This study provides evidence of excess prevalence of early life risk factors for obesity, in particular maternal obesity, poor maternal diet during pregnancy and high weight in infancy in Māori and Pacific infants in New Zealand. Given the striking ethnic inequalities in obesity in childhood and adulthood in New Zealand, this suggests an urgent need for preventive interventions specifically targeting high-risk ethnic groups, including those focusing on pre-pregnancy, pregnancy and early infancy.

## Supplementary Material

Web supplement

## References

[1] Wang Y, Lobstein T Worldwide trends in childhood overweight and obesity. Int J Pediatr Obes 2006;1:11–25. 10.1080/17477160600586747 17902211

[2] Health Mo. New Zealand Health Survey: annual update of key findings 2012/13. http://www.health.govt.nz/publication/new-zealand-health-survey-annual-update-key-findings-2012-13. Secondary New Zealand Health Survey: annual update of key findings 2012/13. http://www.health.govt.nz/publication/new-zealand-health-survey-annual-update-key-findings-2012-13 2013.

[3] Owen CG, Whincup PH, Orfei L, et al Is body mass index before middle age related to coronary heart disease risk in later life? Evidence from observational studies. Int J Obes 2009;33:866–77. 10.1038/ijo.2009.102 PMC2726133 19506565

[4] Hannon TS, Rao G, Arslanian SA Childhood obesity and type 2 diabetes mellitus. Pediatrics 2005;116:473–80. 10.1542/peds.2004-2536 16061606

[5] Russell-Mayhew S, McVey G, Bardick A, et al Mental health, wellness, and childhood overweight/obesity. J Obes 2012;2012:281801 10.1155/2012/281801 22778915 PMC3388583

[6] von Hinke Kessler Scholder S, Davey Smith G, Lawlor DA, et al The effect of fat mass on educational attainment: examining the sensitivity to different identification strategies. Econ Hum Biol 2012;10:405–18. 10.1016/j.ehb.2012.04.015 22709667 PMC3899051

[7] Kipping RR, Jago R, Lawlor DA Obesity in children. Part 1: epidemiology, measurement, risk factors, and screening. BMJ 2008;337:a1824 10.1136/bmj.a1824 18922835

[8] Reilly JJ, Wilson D ABC of obesity. Childhood obesity. BMJ 2006;333 (7580):1207–10. 10.1136/bmj.39048.503750.BE 17158388 PMC1693659

[9] Parsons TJ, Power C, Logan S, et al Childhood predictors of adult obesity: a systematic review. Int J Obes Relat Metab Disord 1999;23 (Suppl 8):S1–107. 10641588

[10] Shrewsbury V, Wardle J Socioeconomic status and adiposity in childhood: a systematic review of cross-sectional studies 1990–2005. Obesity (Silver Spring) 2008;16 (2):275–84. 10.1038/oby.2007.35 18239633

[11] Howe LD, Tilling K, Galobardes B, et al Socioeconomic disparities in trajectories of adiposity across childhood. Int J Pediatr Obes 2011;6:e144–53. 10.3109/17477166.2010.500387 20860432 PMC5102325

[12] Lawlor DA, Relton C, Sattar N, et al Maternal adiposity—a determinant of perinatal and offspring outcomes? Nat Rev Endocrinol 2012;8:679–88. 10.1038/nrendo.2012.176 23007319

[13] Poston L Maternal obesity, gestational weight gain and diet as determinants of offspring long term health. Best Pract Res Clin Endocrinol Metab 2012;26 (5):627–39. 10.1016/j.beem.2012.03.010 22980045

[14] Kim SY, Sharma AJ, Callaghan WM Gestational diabetes and childhood obesity: what is the link? Curr Opin Obstet Gynecol 2012;24 (6):376–81. 10.1097/GCO.0b013e328359f0f4 23000698 PMC4392761

[15] Imai CM, Gunnarsdottir I, Thorisdottir B, et al Associations between infant feeding practice prior to six months and body mass index at six years of age. Nutrients 2014;6:1608–17. 10.3390/nu6041608 24747694 PMC4011054

[16] Jwa SC, Fujiwara T, Kondo N Latent protective effects of breastfeeding on late childhood overweight and obesity: a nationwide prospective study. Obesity (Silver Spring) 2014;22:1527–37. 10.1002/oby.20735 24591416

[17] Ong KK, Ahmed ML, Emmett PM, et al Association between postnatal catch-up growth and obesity in childhood: prospective cohort study. BMJ 2000;320:967–71. 10.1136/bmj.320.7240.967 10753147 PMC27335

[18] Ong KK, Emmett P, Northstone K, et al Infancy weight gain predicts childhood body fat and age at menarche in girls. J Clin Endocrinol Metab 2009;94:1527–32. 10.1210/jc.2008-2489 19240149

[19] Ong KK, Loos RJ Rapid infancy weight gain and subsequent obesity: systematic reviews and hopeful suggestions. Acta paediatr 2006;95 (8):904–8. 10.1080/08035250600719754 16882560

[20] Picot J, Jones J, Colquitt JL, et al The clinical effectiveness and cost-effectiveness of bariatric (weight loss) surgery for obesity: a systematic review and economic evaluation. Health Technol Assess 2009;13:1–190, 215–357, iii–iv 10.3310/hta13410 19726018

[21] Loveman E, Frampton GK, Shepherd J, et al The clinical effectiveness and cost-effectiveness of long-term weight management schemes for adults: a systematic review. Health Technol Assess 2011;15:1–182. 10.3310/hta15020 PMC4781196 21247515

[22] Ara R, Blake L, Gray L, et al What is the clinical effectiveness and cost-effectiveness of using drugs in treating obese patients in primary care? A systematic review. Health Technol Assess 2012;16:iii–xiv, 1–195 10.3310/hta16050 PMC4781292 22340890

[23] Livhits M, Mercado C, Yermilov I, et al Preoperative predictors of weight loss following bariatric surgery: systematic review. Obes Surg 2012;22:70–89. 10.1007/s11695-011-0472-4 21833817

[24] Singh AS, Mulder C, Twisk JW, et al Tracking of childhood overweight into adulthood: a systematic review of the literature. Obes Rev 2008;9:474–88. 10.1111/j.1467-789X.2008.00475.x 18331423

[25] Lang K Measuring ethnicity in the New Zealand population census. Wellington, New Zealand: Statistics New Zealand, 2010.

[26] Rajput N, Tuohy P, Mishra S, et al Overweight and obesity in 4–5-year-old children in New Zealand: results from the first 4 years (2009–2012) of the B4School Check programme. J Paediatr Child Health Published Online First: 26 Aug 2014. doi:10.1111/jpc.12716 10.1111/jpc.12716 25157848

[27] Utter J, Denny S, Crengle S, et al Overweight among New Zealand adolescents: associations with ethnicity and deprivation. Int J Pediatr Obes 2010;5:461–6. 10.3109/17477160903568439 20233145

[28] White P, Gunston J, Salmond C, et al Atlas of Socioeconomic Deprivation in New Zealand NZDep2006. Wellington, New Zealand: Ministry of Health, 2008.

[29] Northstone K, Emmett PM Are dietary patterns stable throughout early and mid-childhood? A birth cohort study. Br J Nutr 2008;100 (5):1069–76. 10.1017/S0007114508968264 18377690 PMC2629612

[30] Northstone K, Emmett PM, Rogers I Dietary patterns in pregnancy and associations with nutrient intakes. Br J Nutr 2008;99 (2):406–15. 17764600 10.1017/S0007114507803977 PMC2492390

[31] Northstone K, Smith AD, Cribb VL, et al Dietary patterns in UK adolescents obtained from a dual-source FFQ and their associations with socio-economic position, nutrient intake and modes of eating. Public Health Nutr 2014;17:1476–85. 10.1017/S1368980013001547 23782861 PMC10282453

[32] Howe LD, Tilling K, Galobardes B, et al Loss to follow-up in cohort studies: bias in estimates of socioeconomic inequalities. Epidemiology 2013;24:1–9. 10.1097/EDE.0b013e31827623b1 23211345 PMC5102324

[33] Spratt M, Carpenter J, Sterne JA, et al Strategies for multiple imputation in longitudinal studies. Am J Epidemiol 2010;172:478–87. 10.1093/aje/kwq137 20616200

[34] Little RJA, Rubin DB Statistical analysis with missing data. 2nd edn Hoboken, New Jersey, USA: John Wiley & Sons, 2002.

[35] Reilly JJ, Armstrong J, Dorosty AR, et al Early life risk factors for obesity in childhood: cohort study. BMJ 2005;330:1357 10.1136/bmj.38470.670903.E0 15908441 PMC558282

[36] Simmons D Relationship between maternal glycaemia and birth weight in glucose-tolerant women from different ethnic groups in New Zealand. Diabet Med 2007;24 (3):240–4. 10.1111/j.1464-5491.2007.02081.x 17263762

[37] Rush E, Gao W, Funaki-Tahifote M, et al Birth weight and growth trajectory to six years in Pacific children. Int J Pediatr Obes 2010;5:192–9. 10.3109/17477160903268290 19878093

[38] Thompson JM, Wall C, Becroft DM, et al Maternal dietary patterns in pregnancy and the association with small-for-gestational-age infants. Br J Nutr 2010;103:1665–73. 10.1017/S0007114509993606 20211035

[39] Benny PS, Benny SC, Sin IL Nutrition in pregnancy in the Wellington region. N Z Med J 1991;104 (905):29–32. 1996186

[40] Kramer MS, Moodie EE, Platt RW Infant feeding and growth: can we answer the causal question? Epidemiology 2012;23:790–4. 10.1097/EDE.0b013e31826cc0e9 23038108

[41] Martin RM, Patel R, Kramer MS, et al Effects of promoting longer-term and exclusive breastfeeding on adiposity and insulin-like growth factor-I at age 11.5 years: a randomized trial. JAMA 2013;309:1005–13. 10.1001/jama.2013.167 23483175 PMC3752893

[42] Brion MJ, Lawlor DA, Matijasevich A, et al What are the causal effects of breastfeeding on IQ, obesity and blood pressure? Evidence from comparing high-income with middle-income cohorts. Int J Epidemiol 2011;40:670–80. 10.1093/ije/dyr020 21349903 PMC3147072

[43] Howe LD, Tilling K, Lawlor DA Accuracy of height and weight data from child health records. Arch Dis Child 2009;94 (12):950–4. 10.1136/adc.2009.162552 19689966

[44] Dekkers JC, van Wier MF, Hendriksen IJ, et al Accuracy of self-reported body weight, height and waist circumference in a Dutch overweight working population. BMC Med Res Methodol 2008;8:69 10.1186/1471-2288-8-69 18957077 PMC2605752

[45] Sharples H, Crutchley PW, Garcia JA, et al Agreement between measured and self-reported height, weight and BMI in predominantly European middle-aged New Zealanders: findings from a nationwide 1989 survey. N Z Med J 2012;125: 60–9. 23178605

[46] Cole TJ, Henson GL, Tremble JM, et al Birthweight for length: ponderal index, body mass index or Benn index? Ann Hum Biol 1997;24:289–98. 10.1080/03014469700005032 9239434

[47] Pietilainen KH, Kaprio J, Rasanen M, et al Tracking of body size from birth to late adolescence: contributions of birth length, birth weight, duration of gestation, parents’ body size, and twinship. Am J Epidemiol 2001;154:21–9. 10.1093/aje/154.1.21 11427401

[48] Salmond CE, Crampton P Development of New Zealand's deprivation index (NZDep) and its uptake as a national policy tool. Can J Public Health 2012; 103:S7–11. 23618071

