# Supplementary material for: Ethnic differences in risk factors for obesity in New Zealand infants
Source: J Epidemiol Community Health. 2015 Mar 20;69(6):516–22. doi: 10.1136/jech-2014-204464 (PMC4453486; doi:10.1136/jech-2014-204464)
Supplement: Web supplement [file jech-2014-204464-s1.pdf]

ONLINE-ONLY SUPPLEMENTARY MATERIAL

Ethnic differences in risk factors for obesity in New Zealand infants

Laura D Howe*[1,2,3], Lis Ellison-Loschmann[3], Neil Pearce[3,4], Jeroen Douwes[3], Mona Jeffreys[2], Ridvan Firestone[3]

1 - MRC Integrative Epidemiology Unit at the University of Bristol, University of Bristol, Bristol, UK

2 - School of Social and Community Medicine, University of Bristol, Bristol, UK

3 - Centre for Public Health Research, Massey University, Wellington, New Zealand

4 - London School of Hygiene and Tropical Medicine, London, UK

* Corresponding author. MRC Integrative Epidemiology Unit at the University of Bristol, Oakfield House, Oakfield Grove, Bristol BS8 2BN, UK. Email: laura.howe@bristol.ac.uk

Supplementary Table 1. Distributions of variables in the observed and imputed data. Values are % or mean (SD)

	Imputed data	Observed data	
		N	
**Socio-demographic variables**			
Child's ethnicity			
European	71.8%	687	71.8%
Māori or Pacific	16.7%		16.7%
Other	11.5%		11.5%
Mother educated to less than degree level	15.8%	544	15.1%
Mother's age			
<25 years	9.1%	545	7.9%
25 to <35 years	63.3%		64.6%
≥ 35 years	27.7%		27.5%
NZ Deprivation Index 2006			
1 (lowest deprivation)	25.9%	544	26.1%
2	28.2%		27.9%
3	19.5%		19.5%
4	16.7%		16.9%
5 (highest deprivation)	9.7%		9.6%
**Maternal, pregnancy and delivery characteristics**			
Maternal pre-pregnancy BMI			
<25 kg/m^2	57.8%	529	58.2%
25 to <30 kg/m^2	27.3%		27.2%
≥30 kg/m^2	15.0%		14.6%
Mother did physical activity pre-pregnancy	82.9%	543	83.4%
Mother's diet in pregnancy*			
Processed foods (SD)	0.0 (1.0)	927	0.0 (1.00)
Snacks (SD)	0.0 (1.0)	927	0.0 (1.00)
Healthy foods (SD)	0.0 (1.0)	927	0.0 (1.00)
Sweet foods (SD)	0.0 (1.0)	927	0.0 (1.00)
Starchy foods (SD)	0.0 (1.0)	927	0.0 (1.00)
Hypertension in pregnancy	12.8%	685	12.9%
Diabetes in pregnancy	3.8%	685	3.8%
**Infancy characteristics**			
Child's weight in first week (kg)	3.52 (0.58)	671	3.52 (0.58)
Child's weight at 3 months (kg)	6.06 (0.89)	636	6.05 (0.89)
Exclusive breastfeeding in first 3 days	79.7%	677	80.2%
Exclusive breastfeeding at 3 months	53.2%	684	53.2%
Solid foods introduced by 3 months	30.0%	682	30.1%

* Dietary pattern scores are standardised to have a mean of zero and variance of one; values in this table can therefore be interpreted as standard deviations from the overall population mean

Supplementary Table 2. Characteristics of participants. Values are mean (SD) or number (%)

	Participants with data on all variables N=422	Participants not included in complete case analysis due to missing data on one or more variables		P value*
		% with missing data on this variable		
**Socio-demographic variables**				
Child's ethnicity				
European	307 (72.8%)	22%	186 (70.2%)	
Māori or Pacific	64 (15.2%)		51 (19.3%)	
Other	51 (12.1%)		28 (10.6%)	0.35
Mother educated to less than degree level	60 (14.2%)	52%	118 (19.3%)	0.03
Mother's age				
<25 years	31 (7.4%)	52%	84 (13.6%)	
25 to <35 years	277 (65.6%)		359 (58.2%)	
≥ 35 years	114 (27.0%)		174 (28.2%)	0.004
NZ Deprivation Index 2006				
1 (lowest deprivation)	109 (25.8%)	52%	167 (27.1%)	
2	118 (28.0%)		127 (20.6%)	
3	84 (19.9%)		144 (23.4%)	
4	73 (17.3%)		103 (16.7%)	
5 (highest deprivation)	38 (9.0%)		75 (12.2%)	0.05
**Maternal, pregnancy and delivery characteristics**				
Maternal pre-pregnancy BMI				
<25 kg/m²	245 (58.1%)	49%	331 (56.8%)	
25 to <30 kg/m²	113 (26.8%)		170 (29.2%)	
≥30 kg/m²	64 (15.2%)		82 (14.1%)	0.68
Mother did physical activity pre-pregnancy**	354 (83.9%)	52%	506 (82.1%)	0.46
Mother's diet in pregnancy				
Processed foods (SD)	-0.04 (1.02)	43%	0.03 (1.02)	0.25
Snacks (SD)	-0.06 (1.00)	43%	0.05 (1.00)	0.08
Healthy foods (SD)	0.07 (1.00)	43%	-0.06 (1.00)	0.04
Sweet foods (SD)	0.00 (0.98)	43%	0.00 (1.02)	0.97
Starchy foods (SD)	-0.01 (1.01)	43%	0.01 (0.99)	0.77
Hypertension in pregnancy	50 (11.9%)	22%	38 (14.6%)	0.32
Diabetes in pregnancy	15 (3.6%)	22%	11 (4.2%)	0.68
**Infancy characteristics**				
Child's weight in first week (kg)	3.54 (0.56)	21%	3.49 (0.60)	0.27
Child's weight at 3 months (kg)	6.05 (0.89)	18%	6.07 (0.88)	0.74
Exclusive breastfeeding in first 3 days	340 (80.6%)	22%	203 (79.6%)	0.76
Exclusive breastfeeding at 3 months	225 (53.3%)	22%	139 (53.1%)	0.95
Solid foods introduced by 3 months	125 (29.6%)	22%	80 (30.8%)	0.75

*P value testing the null hypothesis that the mean or percentage does not differ between those included in analyses and those excluded due to missing data; p values are generated from t-tests or chi-squared tests
** Dietary pattern scores are standardised to have a mean of zero and variance of one; values in this table can therefore be interpreted as standard deviations from the overall population mean

Supplementary Table 3. Principal component analysis of dietary data; first five principal components

Values are ‘eigenvectors’ from rotated principal components, which can be interpreted as weights assigned to the food items within each dietary pattern. For example in the ‘high fat, low fruit & veg, high sugar’ dietary pattern, Pies has a large positive weight (0.2660) whereas fresh fruit has a low negative weight (-0.1158). Therefore the more often a woman reports eating pies, the higher her score for the ‘high fat, low fruit & veg, high sugar’ dietary pattern will be, and vice versa for fresh fruit.

	Processed	***Snacks***	***Healthy foods***	***Sweet foods***	***Starchy foods***
Proportion of variance explained	***0.10***	***0.08***	***0.07***	***0.07***	***0.06***
**Boiled veg**	-0.0550	-0.0904	-0.0589	0.0755	-0.0248
**Stir fry veg**	-0.1217	0.2198	0.2764	-0.2163	0.2529
**Frozen veg**	0.0911	0.0129	0.0001	-0.0106	-0.0811
**Fresh fruit**	0.0679	0.0136	0.3655	-0.0234	0.1058
**Oats**	-0.0790	0.0568	0.3979	0.1189	-0.0566
**European bread**	0.0317	0.0371	-0.4535	0.0586	0.0806
**Brown bread**	0.0881	-0.0550	0.4737	0.0108	-0.1140
**Pulses**	0.0253	0.0367	0.3548	-0.0153	0.1480
**Cheese**	0.2632	-0.2813	0.1399	0.3055	-0.0519
**Pies**	0.3346	0.0332	-0.0116	-0.0698	0.0504
**Sausages**	0.1386	-0.0734	-0.0678	0.0224	0.0755
**Burgers**	0.3757	0.0220	0.0536	-0.0711	-0.0577
**Pizza**	0.4259	-0.0897	0.0468	0.0795	-0.0322
**Chips**	0.4604	0.0833	0.0001	-0.0459	-0.0200
**Bbq foods**	-0.0287	-0.0053	0.0629	0.0455	0.0251
**Fried foods**	0.3338	0.0018	-0.0840	0.0254	0.1339
**Rice**	-0.0756	0.0559	-0.0245	-0.0691	0.6786
**Pasta**	0.1135	-0.1401	-0.0166	0.1475	0.5898
**Pudding**	0.0112	0.0033	-0.0354	0.4921	0.0070
**Biscuits**	-0.0365	0.1113	-0.0066	0.4777	0.0216
**Cake**	-0.0372	0.1240	-0.0012	0.4789	0.0338
**Chocolate bars**	-0.0032	0.4619	-0.0749	0.1158	-0.0653
**Chocolate**	-0.0133	0.4698	0.1081	0.1568	-0.0180
**Sweets**	0.0388	0.4489	-0.0015	0.0042	-0.0318
**Crips**	0.1951	0.2895	-0.0607	-0.0494	0.1189
**Fizzy drinks**	0.2126	0.2514	-0.0934	-0.2176	-0.0952

Supplementary Table 4. Association between child's ethnicity and socio-demographic variables in participants with complete data on all variables. N=422
Values are odds ratios (95% confidence intervals) comparing Māori or Pacific children and Other Ethnicity children to European children.

	European N=307	Māori or Pacific N=64	Other N=51
Mother educated to less than degree level	1 (ref)	1.67 (0.84 to 3.28) P=0.13	0.24 (0.06 to 1.04) P=0.06
Mother's age			
<25 years	1 (ref)	2.65 (1.18 to 5.98) P=0.02	0.28 (0.04 to 2.16) P=0.22
25 to <35 years	1 (ref)	1 (ref)	1 (ref)
≥ 35 years	1 (ref)	0.50 (0.24 to 1.05) P=0.07	0.76 (0.39 to 1.50) P=0.43
NZ Deprivation Index 2006			
1 (lowest deprivation)	1 (ref)	1 (ref)	1 (ref)
2	1 (ref)	2.05 (0.83 to 5.08) P=0.12	2.46 (1.02 to 5.95) P=0.05
3	1 (ref)	2.48 (0.97 to 6.33) P=0.06	1.91 (0.71 to 5.10) P=0.20
4	1 (ref)	5.81 (2.38 to 14.18) P<0.001	2.77 (1.02 to 7.51) P=0.05
5 (highest deprivation)	1 (ref)	3.13 (1.04 to 9.44) P=0.04	2.24 (0.67 to 7.41) P=0.19

Supplementary Table 5. Association between child's ethnicity and known risk factors for later obesity in participants with complete data on all variables (unadjusted). N=422

Values are odds ratios or mean differences (95% confidence intervals) comparing Māori or Pacific children and Other Ethnicity children to European children.

	European N=307	Māori or Pacific N=64	Other N=51
**Categorical risk factors; odds ratios and 95% confidence intervals**			
Maternal pre-pregnancy BMI			
<25 kg/m²	1 (ref)	1 (ref)	1 (ref)
25 to <30 kg/m²	1 (ref)	1.28 (0.67 to 2.44) P=0.46	1.32 (0.69 to 2.53) P=0.40
≥30 kg/m²	1 (ref)	2.16 (1.09 to 4.29) P=0.03	0.56 (0.19 to 1.66) P=0.30
Mother did physical activity pre-pregnancy	1 (ref)	1.28 (0.59 to 2.74) P=0.53	1.56 (0.64 to 3.86) P=0.33
Hypertension in pregnancy	1 (ref)	1.59 (0.74 to 3.43) P=0.24	1.60 (0.69 to 3.70) P=0.27
Diabetes in pregnancy	1 (ref)	0.87 (0.19 to 4.01) P=0.86	1.10 (0.24 to 5.11) P=0.91
Exclusive breastfeeding in first 3 days	1 (ref)	0.95 (0.49 to 1.86) P=0.89	1.13 (0.52 to 2.46) P=0.75
Exclusive breastfeeding at 3 months	1 (ref)	0.98 (0.57 to 1.68) P=0.94	1.55 (0.84 to 2.85) P=0.16
Solid foods introduced by 3 months	1 (ref)	1.42 (0.80 to 2.50) P=0.23	1.05 (0.55 to 2.02) P=0.88
**Continuous risk factors; mean differences and 95% confidence intervals**			
Mother's diet in pregnancy*			
Processed foods (SD)	0 (ref)	0.10 (-0.16 to 0.37) P=0.44	-0.10 (-0.39 to 0.18) P=0.48
Snacks (SD)	0 (ref)	0.25 (-0.02 to 0.52) P=0.07	-0.18 (-0.48 to 0.11) P=0.22
Healthy foods (SD)	0 (ref)	-0.06 (-0.33 to 0.21) P=0.65	0.09 (-0.21 to 0.39) P=0.56
Sweet foods (SD)	0 (ref)	-0.29 (-0.56 to -0.03) P=0.03	-0.01 (-0.30 to 0.28) P=0.94
Starchy foods (SD)	0 (ref)	0.23 (-0.04 to 0.50) P=0.10	0.40 (0.10 to 0.70) P=0.01
Child's weight in first week (kg)	0 (ref)	0.04 (-0.12 to 0.19) P=0.64	-0.11 (-0.27 to 0.06) P=0.21
Child's weight at 3 months (kg)	0 (ref)	0.13 (-0.11 to 0.37) P=0.30	0.04 (-0.23 to 0.30) P=0.79

* Dietary pattern scores are standardised to have a mean of zero and variance of one; values in this table can therefore be interpreted as standard deviations from the overall population mean

Supplementary Table 6. Association between child's ethnicity and known risk factors for later obesity in participants with complete data on all variables (with adjustment for socio-demographic characteristics). N=422

Values are odds ratios or mean differences (95% confidence intervals) comparing Māori or Pacific children and Other Ethnicity children to European children.

	European N=307	Māori or Pacific N=64	Other N=51
**Categorical risk factors; odds ratios and 95% confidence intervals**			
Maternal pre-pregnancy BMI			
<25 kg/m²	1 (ref)	1 (ref)	1 (ref)
25 to <30 kg/m²	1 (ref)	1.22 (0.62 to 2.38) P=0.56	1.33 (0.68 to 2.57) P=0.40
≥30 kg/m²	1 (ref)	2.06 (0.99 to 4.28) P=0.05	0.57 (0.19 to 1.73) P=0.32
Mother did physical activity pre-pregnancy	1 (ref)	1.79 (0.79 to 4.06) P=0.17	1.57 (0.63 to 3.94) P=0.34
Hypertension in pregnancy	1 (ref)	1.71 (0.76 to 3.82) P=0.19	1.63 (0.69 to 3.84) P=0.26
Diabetes in pregnancy	1 (ref)	0.87 (0.18 to 4.25) P=0.86	1.06 (0.22 to 5.08) P=0.94
Exclusive breastfeeding in first 3 days	1 (ref)	0.93 (0.46 to 1.87) P=0.84	1.18 (0.54 to 2.58) P=0.68
Exclusive breastfeeding at 3 months	1 (ref)	0.98 (0.56 to 1.72) P=0.95	1.52 (0.82 to 2.82) P=0.19
Solid foods introduced by 3 months	1 (ref)	1.46 (0.80 to 2.64) P=0.22	1.16 (0.60 to 2.25) P=0.66
**Continuous risk factors; mean differences and 95% confidence intervals**			
Mother's diet in pregnancy*			
Processed foods (SD)	0 (ref)	0.02 (-0.25 to 0.29) P=0.89	-0.12 (-0.41 to 0.17) P=0.42
Snacks (SD)	0 (ref)	0.24 (-0.03 to 0.52) P=0.08	-0.22 (-0.51 to 0.08) P=0.15
Healthy foods (SD)	0 (ref)	-0.01 (-0.29 to 0.27) P=0.92	0.09 (-0.21 to 0.39) P=0.55
Sweet foods (SD)	0 (ref)	-0.26 (-0.53 to 0.01) P=0.06	-0.03 (-0.32 to 0.26) P=0.82
Starchy foods (SD)	0 (ref)	0.26 (-0.02 to 0.54) P=0.07	0.38 (0.08 to 0.68) P=0.01
Child's weight in first week (kg)	0 (ref)	0.03 (-0.13 to 0.19) P=0.73	-0.09 (-0.26 to 0.08) P=0.28
Child's weight at 3 months (kg)	0 (ref)	0.11 (-0.14 to 0.36) P=0.40	0.05 (-0.22 to 0.32) P=0.73

* Dietary pattern scores are standardised to have a mean of zero and variance of one; values in this table can therefore be interpreted as standard deviations from the overall population mean

Supplementary Table 7. Association between ethnicity and infant weight at age 3 months, and the degree to which this is explained by other risk factors in participants with complete data on all variables. N=422

Values are mean differences (95% confidence intervals) in infant weight at age 3 months (kg) from linear regression, comparing each ethnic group with the reference category, European ethnicity

	Mean difference (95% CI) in infant weight at 3 months (kg)		
	European<br>N=493	Māori or Pacific<br>N=115	Other<br>N=79
Unadjusted	0 (ref)	0.13 (-0.11 to 0.37)<br>P=0.30	0.04 (-0.23 to 0.30)<br>P=0.79
*Adjusted for socio-demographic variables**	0 (ref)	0.11 (-0.14 to 0.36)<br>P=0.40	0.05 (-0.22 to 0.32)<br>P=0.73
Adjusted for maternal pre-pregnancy BMI	0 (ref)	0.12 (-0.12 to 0.36)<br>P=0.34	0.04 (-0.22 to 0.31)<br>P=0.76
*Adjusted for maternal dietary pattern scores***	0 (ref)	0.14 (-0.10 to 0.39)<br>P=0.25	0.02 (-0.25 to 0.29)<br>P=0.89
*Adjusted for all measured potential mediators****	0 (ref)	0.09 (-0.16 to 0.33)<br>P=0.50	0.02 (-0.26 to 0.27)<br>P=0.99

* Adjusted for maternal education, maternal age and NZ deprivation index
** Adjusted for scores for all five dietary patterns
*** adjusted for maternal education, maternal age, NZ deprivation index, maternal pre-pregnancy BMI, scores for all five dietary patterns, hypertension during pregnancy, diabetes during pregnancy, physical activity during pregnancy, exclusive breastfeeding at 3 days, exclusive breastfeeding at 3 months, and early introduction of solid foods

Supplementary Figure 1. Screeplot of Eigenvalues after PCA of dietary data

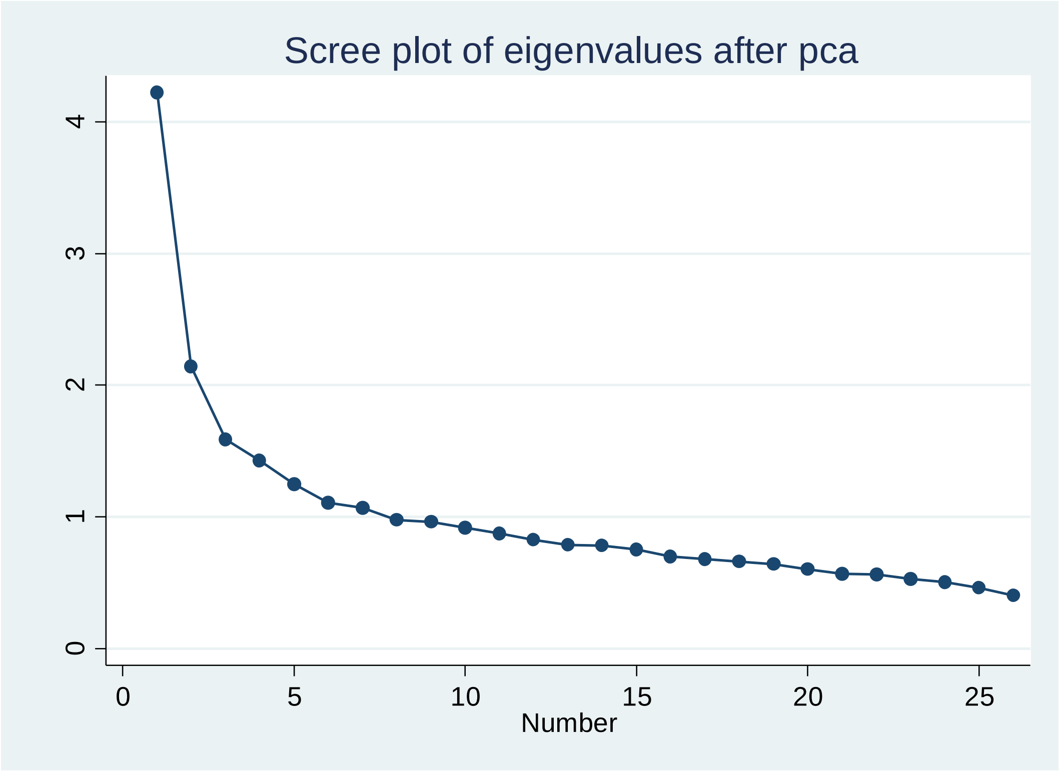

Dietary intake questionnaire used in the ELF study

Diet

7.06 Since becoming pregnant, how many times a week do you eat?

	Never or rarely	Once in 2 weeks	1-3 times a week	4-7 times a week	More than once a day
Poultry<br>EXAMPLE: *Chicken, turkey*	☐	☐	☐	☐	☐
Eggs	☐	☐	☐	☐	☐
Liver, pate, kidney, heart	☐	☐	☐	☐	☐
White fish<br>EXAMPLE: *Cod, fish fingers*	☐	☐	☐	☐	☐
Other Fish<br>EXAMPLE: *Tuna, mackerel*	☐	☐	☐	☐	☐
Shellfish	☐	☐	☐	☐	☐

7.06 (a) Do you eat the fat on meat?

Yes, all of it	☐
Yes, some of it	☐
No	☐
Never eat meat	☐

7.07 Since becoming pregnant what sort of 'spreads' and 'sauces' do you use?

	Yes	No
Butter	☐	☐
Unsalted butter	☐	☐
Polyunsaturated margarine<br>EXAMPLE: *Flora, sunflower*	☐	☐
Butter and margarine blend	☐	☐
Olive oil spread<br>EXAMPLE: *Olivio*	☐	☐
Low salt margarine	☐	☐
Reduced fat margarines	☐	☐
Nutella	☐	☐
Jam/honey	☐	☐
Marmite/vegemite	☐	☐
Peanut butter	☐	☐
Mayonnaise/salad dressing	☐	☐
Tomato sauce/ketchup	☐	☐
Gravy	☐	☐
Other spreads/sauces	☐	☐

Please describe ____

7.08 Since becoming pregnant what sort of fat do you mainly use for <u>frying</u>, <u>cooking</u> or <u>BBQ</u>?

	Yes	No
Butter, dripping, lard, solid cooking fat	☐	☐
Polyunsaturated margarine<br>EXAMPLE: *Flora, Sunflower, Olivio*	☐	☐
Sesame seed oil	☐	☐
Sunflower, soya, corn, olive oil	☐	☐
Other vegetable oil	☐	☐
Other	☐	☐

Please describe ____

Vegetables & Fruit

7.09 Since becoming pregnant, how many times a week do you eat?

	Never or rarely	Once in 2 weeks	1-3 times a week	4-7 times a week	More than once a day
Baked beans	☐	☐	☐	☐	☐
Peas, sweetcorn, broad beans	☐	☐	☐	☐	☐
Cabbage, brussel sprouts, kale & other green leafy vegetables	☐	☐	☐	☐	☐
Other green vegetables<br>EXAMPLE: *Cauliflower, runner beans, leeks*	☐	☐	☐	☐	☐
Carrots	☐	☐	☐	☐	☐
Potatoes	☐	☐	☐	☐	☐

Other root vegetables<br>EXAMPLE: *Turnip, swede, parsnip*	☐	☐	☐	☐	☐
Salad<br>EXAMPLE: *Lettuce, tomatoes, cucumber, etc*	☐	☐	☐	☐	☐
Boiled vegetables	☐	☐	☐	☐	☐
Stir-fried vegetables	☐	☐	☐	☐	☐
Frozen vegetables	☐	☐	☐	☐	☐
Processed fruit	☐	☐	☐	☐	☐
Frozen fruit	☐	☐	☐	☐	☐
Fresh fruit<br>EXAMPLE: *Apple, pear, banana, orange, grapes etc*	☐	☐	☐	☐	☐
Other fruit/veges not listed	☐	☐	☐	☐	☐

Grains

7.10 Since becoming pregnant, how many times a week do you eat?

	Never or rarely	Once in 2 weeks	1-3 times a week	4-7 times a week	More than once a day
Oat cereals<br>EXAMPLE: *Porridge, muesli*	☐	☐	☐	☐	☐
Wholegrain/bran cereals<br>EXAMPLE: *Weetbix, All Bran, Bran Flakes, Fruit & Fibre*	☐	☐	☐	☐	☐
Other cereals<br>EXAMPLE: *Cornflakes, Rice bubbles, Special K*	☐	☐	☐	☐	☐

7.11 **Since becoming pregnant, how many pieces of bread, rolls, chappatis & pita bread do you eat on a usual day?**

Less than 1 ☐

1-2 ☐

3-4 ☐

5 or more ☐

7.12 **What types of bread do you eat most days?**

☞ ***Multiple answers possible***

	Yes	No	Don't know
White bread	☐	☐	☐
Brown/granary bread	☐	☐	☐
Wholemeal bread	☐	☐	☐
Chappatis, Nan bread	☐	☐	☐
Other types	☐	☐	☐
Please describe ______________________________			
Don't usually eat any bread	☐	☐	☐

Legumes

7.13 Since becoming pregnant, how many times a week do you eat?

	Never or rarely	Once in 2 weeks	1-3 times a week	4-7 times a week	More than once a day
Pulses<br>EXAMPLE: *Dried beans, lentils*	☐	☐	☐	☐	☐
Nuts, nut roast	☐	☐	☐	☐	☐
Bean curd (tofu)	☐	☐	☐	☐	☐
Tahini<br>EXAMPLE: *Used to make hummus, almond butter, peanut butter*	☐	☐	☐	☐	☐
Soya meat, vegeburgers	☐	☐	☐	☐	☐
Other legumes not listed	☐	☐	☐	☐	☐

Dairy Food

7.14 Since becoming pregnant, how often have you eaten the following?

	Never	Less than once a week	1-6 times a week	More than 6 times a week	Don't know
Yoghurt (Full)	☐	☐	☐	☐	☐
Yoghurt (Lite)	☐	☐	☐	☐	☐
Yoghurt (Low fat)	☐	☐	☐	☐	☐
Cream (Full)	☐	☐	☐	☐	☐
Cream (Lite)	☐	☐	☐	☐	☐
Cream Cheese (Full)	☐	☐	☐	☐	☐
Cheese<br>EXAMPLE: *Edam, mozzarella*	☐	☐	☐	☐	☐

7.15 Do you drink milk?

Yes ☐ → **IF *'YES'* PLEASE GO TO QUESTION 7.15(a)**

No ☐ → **IF *'NO'* PLEASE GO TO QUESTION 7.16**

7.15 (a) **What type of milks do you drink?**

	Usually	Some times	Not at all	Don't know
Silver top (Full cream)	☐	☐	☐	☐
Blue top (Common)	☐	☐	☐	☐
Light Blue top (Lite)	☐	☐	☐	☐
Dark Green top (Super trim)	☐	☐	☐	☐
Orange top (Vitamin A & D)	☐	☐	☐	☐
Yellow top (Extra calcium)	☐	☐	☐	☐
Purple top (Omega 3 & Vitamin E)	☐	☐	☐	☐
Soya milk	☐	☐	☐	☐
Rice milk	☐	☐	☐	☐
Goat/sheep milk	☐	☐	☐	☐
Milk powder	☐	☐	☐	☐
Other	☐	☐	☐	☐

7.16 **Since becoming pregnant, do you drink fresh milk directly from a farm?**

Yes	☐	→	**IF *'YES'* PLEASE GO TO QUESTION 7.16(a)**
No	☐	→	**IF *'NO'* PLEASE GO TO QUESTION 7.17**

7.16 (a) **Do you normally boil this milk?**

Yes ☐

No ☐

7.16 (b) **Since becoming pregnant, how <u>many glasses</u> of fresh milk directly from a farm have you drunk?**

☐ Glass per week

Other Food

7.17 **Since becoming pregnant, how many times a week do you eat?**

	Never or rarely	Once in 2 weeks	1-3 times a week	4-7 times a week	More than once a day
Pies, pasties<br>EXAMPLE: *Pork, steak, meat*	☐	☐	☐	☐	☐
Sausages	☐	☐	☐	☐	☐
Hamburgers	☐	☐	☐	☐	☐
Pizza	☐	☐	☐	☐	☐
Chips/fries	☐	☐	☐	☐	☐
BBQ food	☐	☐	☐	☐	☐
Fried food<br>EXAMPLE: *Fried fish, eggs, bacon, chops etc*	☐	☐	☐	☐	☐
Roast potatoes (Cooked in fat)	☐	☐	☐	☐	☐
Boiled/mashed potatoes	☐	☐	☐	☐	☐
Rice (Boiled)	☐	☐	☐	☐	☐
Pasta	☐	☐	☐	☐	☐

EXAMPLE: *Spaghetti, noodles*

	Never or rarely	Once in 2 weeks	1-3 times a week	4-7 times a week	More than once a day
Other food not listed	☐	☐	☐	☐	☐

Snacks

7.18 Since becoming pregnant, how many times a week do you eat?

	**Never or rarely**	**Once in 2 weeks**	**1-3 times a week**	**4-7 times a week**	**More than once a day**
Pudding	☐	☐	☐	☐	☐
EXAMPLE: *Fruit pie, crumble, cheese-cake, mousse, gateaux*					
Biscuits	☐	☐	☐	☐	☐
EXAMPLE: *Digestives, shortbread, gingernuts, chocolate biscuits*					
Cripbreads	☐	☐	☐	☐	☐
EXAMPLE: *Ryvita, cabin bread*					
Cakes or buns	☐	☐	☐	☐	☐
EXAMPLE: *Fruit cake, sponge, teacake, buns, doughnuts, scones, custard pie*					
Chocolate bars	☐	☐	☐	☐	☐
EXAMPLE: *Mars, Twix, Moro, Picnic, Bounty, Crunchie*					
Chocolate	☐	☐	☐	☐	☐
EXAMPLE: *Dairy milk, plain, dark, nut, fruit, filled*					
Sweets	☐	☐	☐	☐	☐
EXAMPLE: *Boiled lollies, toffees*					
Crisps/potato chips	☐	☐	☐	☐	☐
Other snacks not listed	☐	☐	☐	☐	☐

7.19 How <u>many times a week</u> do you eat take-away foods for your main meals?

Never or rarely	☐
1-2	☐
3-4	☐
5 or more	☐
10 or more	☐

Seasoning

7.20 When you are eating, do you regularly add:

	**Never**	**Rarely**	**Often**	**Always**
Pepper	☐	☐	☐	☐
Salt (Table)	☐	☐	☐	☐
Salt (Rock)	☐	☐	☐	☐
Cheese	☐	☐	☐	☐
Oil	☐	☐	☐	☐
Soya sauces	☐	☐	☐	☐
Other sauces	☐	☐	☐	☐
Other spices/condiments	☐	☐	☐	☐

EXAMPLE: *Brewer's yeast, sea kelp granules, fresh herbs*

Drinks

7.21 Since becoming pregnant, how many times a week do you drink?

	Never or rarely	Once in 2 weeks	1-3 times a week	4-7 times a week	More than once a day
Fizzy drinks (In a can)<br>EXAMPLE: *L&P, Sprite, Coca Cola*	☐	☐	☐	☐	☐
Fizzy drinks (In a bottle)<br>EXAMPLE: *L&P, Sprite, Coca Cola*	☐	☐	☐	☐	☐
Bottled juice<br>EXAMPLE: *Keri juice*	☐	☐	☐	☐	☐
Juice in a carton<br>EXAMPLE: *Just Juice, Fresh up, Ribena*	☐	☐	☐	☐	☐
Water	☐	☐	☐	☐	☐
Water (Carbonated)<br>EXAMPLE: *Spring water, H_2O sparkling drink*	☐	☐	☐	☐	☐
Tea	☐	☐	☐	☐	☐
Herbal teas (Non caffeine)	☐	☐	☐	☐	☐
Coffee	☐	☐	☐	☐	☐
Other drinks not listed	☐	☐	☐	☐	☐

7.22 Do you regularly drink tea?

Yes ☐ → **IF *'YES'* PLEASE GO TO QUESTION 7.22(a)**

No ☐ → **IF *'NO'* PLEASE GO TO QUESTION 7.23**

7.22 (a) **How many <u>cups of tea</u> do you drink in a day?**

☞ ***Do not include Herbal teas***

☐ Cups

7.22 (b) **How many <u>cups of tea</u> you drink each day are decaffeinated?**

☐ Cups

7.23 **Do you regularly drink coffee?**

Yes ☐ → **IF *'YES'* PLEASE GO TO QUESTION 7.23(a)**

No ☐ → **IF *'NO'* PLEASE GO TO QUESTION 7.24**

7.23 (a) **How many <u>cups of decaffeinated coffee</u> do you drink in a day?**

☐ Cups

7.23 (b) **How many <u>cups of instant coffee</u> do you drink each day?**

☐ Cups

7.23 (c) **How many <u>cups of real coffee</u> do you drink each day?**

☐ Cups

7.24 How many teaspoons or honey or sugar do you add to hot drinks?

Never or rarely ☐

1-2 ☐

3-4 ☐

5 or more ☐

7.25 Do you drink Herbal teas at all?

Yes, sometimes or always ☐ → **IF *'YES'* PLEASE GO TO QUESTION 7.25(a)**

No, not at all ☐ → **IF *'NO'* PLEASE GO TO QUESTION 7.26**

7.25 (a) What do you drink?

☞ ***Give exact name if you can***

____

____

7.25 (b) In which Trimester period?

☞ ***Multiple answers possible***

1st Trimester (ie 1st 3 months) ☐

2nd Trimester (ie 2nd 3 months) ☐

3rd Trimester (ie 3rd 3 months) ☐

**7.25 (c)**	**How many times a day did you drink this tea?**
☐	Times
